# Hypoxia impacts human MSC response to substrate stiffness during chondrogenic differentiation

**DOI:** 10.1016/j.actbio.2019.03.002

**Published:** 2019-04-15

**Authors:** Daniel A. Foyt, Dheraj K. Taheem, Silvia A. Ferreira, Michael D.A. Norman, Jonna Petzold, Gavin Jell, Agamemnon E. Grigoriadis, Eileen Gentleman

**Affiliations:** a Centre for Craniofacial and Regenerative Biology, King’s College London, London SE1 9RT, UK; b Division of Surgery & Interventional Science, University College London, London NW3 2PF, UK

**Keywords:** Cartilage, Tissue engineering, Hypoxia, Mechanotransduction, Mesenchymal stem cell

## Abstract

Tissue engineering strategies often aim to direct tissue formation by mimicking conditions progenitor cells experience within native tissues. For example, to create cartilage *in vitro*, researchers often aim to replicate the biochemical and mechanical milieu cells experience during cartilage formation in the developing limb bud. This includes stimulating progenitors with TGF-β_1/3_, culturing under hypoxic conditions, and regulating mechanosensory pathways using biomaterials that control substrate stiffness and/or cell shape. However, as progenitors differentiate down the chondrogenic lineage, the pathways that regulate their responses to mechanotransduction, hypoxia and TGF-β may not act independently, but rather also impact one another, influencing overall cell response. Here, to better understand hypoxia’s influence on mechanoregulatory-mediated chondrogenesis, we cultured human marrow stromal/mesenchymal stem cells (hMSC) on soft (0.167 kPa) or stiff (49.6 kPa) polyacrylamide hydrogels in chondrogenic medium containing TGF-β_3_. We then compared cell morphology, phosphorylated myosin light chain 2 staining, and chondrogenic gene expression under normoxic and hypoxic conditions, in the presence and absence of pharmacological inhibition of cytoskeletal tension. We show that on soft compared to stiff substrates, hypoxia prompts hMSC to adopt more spread morphologies, assemble in compact mesenchymal condensation-like colonies, and upregulate *NCAM* expression, and that inhibition of cytoskeletal tension negates hypoxia-mediated upregulation of molecular markers of chondrogenesis, including *COL2A1* and *SOX9*. Taken together, our findings support a role for hypoxia in regulating hMSC morphology, cytoskeletal tension and chondrogenesis, and that hypoxia’s effects are modulated, at least in part, by mechanosensitive pathways. Our insights into how hypoxia impacts mechanoregulation of chondrogenesis in hMSC may improve strategies to develop tissue engineered cartilage.

**Statement of Significance:**

Cartilage tissue engineering strategies often aim to drive progenitor cell differentiation by replicating the local environment of the native tissue, including by regulating oxygen concentration and mechanical stiffness. However, the pathways that regulate cellular responses to mechanotransduction and hypoxia may not act independently, but rather also impact one another. Here, we show that on soft, but not stiff surfaces, hypoxia impacts human MSC (hMSC) morphology and colony formation, and inhibition of cytoskeletal tension negates the hypoxia-mediated upregulation of molecular markers of chondrogenesis. These observations suggest that hypoxia’s effects during hMSC chondrogenesis are modulated, at least in part, by mechanosensitive pathways, and may impact strategies to develop scaffolds for cartilage tissue engineering, as hypoxia’s chondrogenic effects may be enhanced on soft materials.

## Graphical abstract

**Figure:**
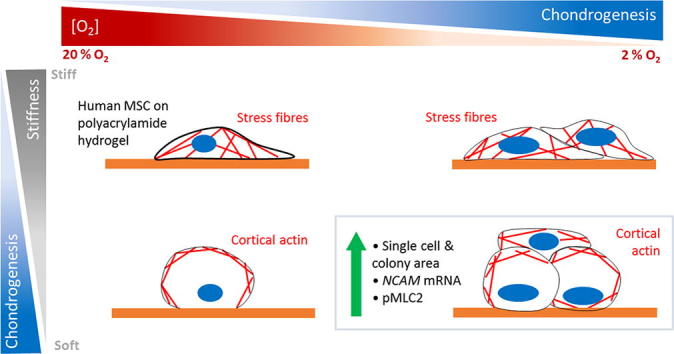

## 1 Introduction

Osteoarthritis (OA) is one of the leading causes of disability worldwide and constitutes a significant individual and socioeconomic burden [1]. Indeed, the costs of OA in the USA, Canada, UK, France and Australia may account for between 1 and 2.5% of these countries’ gross domestic products [2]. One goal in the field of cartilage tissue engineering (TE) is to repair cartilage lesions before they progress to OA. To coax progenitor cells to differentiate appropriately and produce cartilage, many TE strategies aim to create scaffolds that mimic characteristics of the native tissue that progenitor cells are exposed to as the tissue is formed [3], [4], [5]. For cartilage, which has a very poor capacity for self-repair in the adult, much of what is known about the conditions that foster cartilage formation come from developmental studies, including the study of limb bud development and endochondral ossification in the axial skeleton. Cartilage TE strategies that mimic both the biochemical milieu as well as the mechanical environment that native cells experience during these developmental processes may yield engineered constructs that can repair damaged adult tissues [6], [7], [8], [9].

Hypoxia, whose cellular response is mediated via the hypoxia inducible factor (HIF) pathway, plays central roles in regulating chondrogenesis and maintaining the articular chondrocyte phenotype throughout life [10], [11], [12], [13]. Oxygen concentration gradients in the developing limb bud, which are maintained in adult osteochondral tissue, are known to contribute to the formation of cartilage, and are mediated, at least in part, through the activity of HIF [14]. Conditional deletion of HIF-1α, the oxygen-responsive subunit of the HIF complex, during limb development induces loss of the cartilage growth plate via a combination of cell apoptosis and perturbations to normal SOX9-mediated chondrocyte proliferation [11], [14], [15]. HIF-1α has similarly been shown to play a central role in the differentiation of mesenchymal progenitors to chondrocytes by binding to the SOX9 promoter region, thereby activating expression of the master transcriptional regulator of chondrogenesis and its downstream targets [12].

However, hypoxia is not the only characteristic of the native milieu that is known to drive chondroprogenitor commitment. There is also evidence that chondrogenic lineage specification is regulated by the physical environment of the condensing mesenchyme. Condensation of the mesenchyme in the limb bud is required to initiate chondrogenesis in the developing growth plate [16], and physical stimuli including round cell morphologies, a cortical actin network, and mechanically soft environments all appear to promote this process [17], [18]. Indeed, chondrocytes cultured on soft polyacrylamide (PA) hydrogels maintain a more chondrocyte-like phenotype than cells grown on stiff PA hydrogels, which express markers typical of hypertrophic chondrocytes [19], [20]. Moreover, marrow stromal/mesenchymal stem cells (MSC) with cortical actin arrangements express chondrogenic markers at higher levels than those with spread morphologies that produce defined stress fibres [18].

The round cell morphologies in the condensing mesenchyme and later in native articular cartilage are reminiscent of other mesenchymal cells that also adopt round morphologies (particularly on soft substrates), such as adipocytes. However, whereas adipocytes’ round morphologies have been associated with low levels of cytoskeletal tension [21], during chondrogenesis, cytoskeletal tension appears to be an important driver of mesenchymal condensation [22]. Indeed, there is evidence that the Ras Homolog Family Member A (RhoA)/Rho-associated protein kinase (ROCK) pathway, which is known to play important roles in regulating cytoskeletal tension [23], [24], may directly regulate chondrogenesis [25], [26], and that this process appears to be dependent on the mechanical environment. For example, RhoA/ROCK inhibition reduces chondrogenesis in ATDC5 cells on soft substrates; but on stiff substrates, ROCK inhibition increases expression of markers of articular cartilage, including *SOX9*, *COL2A1* and *ACAN* [17]. Moreover, when human MSC (hMSC) in which a constitutively activated RhoA was overexpressed were cultured on soft substrates, they upregulated expression of *COL2A1* [27]. RhoA/ROCK’s role in chondrogenesis has also been observed *in vivo* where ROCK-mediated cytoskeletal tension protects mice from developing OA following surgical destabilisation of the medial meniscus [20]. These observations suggest that in an appropriate mechanical environment, ROCK activity stimulates chondrogenesis, and are in contrast to the role of RhoA/ROCK signalling in hMSC adipogenesis, where differentiation is highly dependent on its suppression [21].

Taken together, these observations suggest that factors which maintain ROCK-mediated actin-myosin tension, but also maintain round cell morphologies, similarly to the conditions that progenitor cells experience during mesenchymal condensation in the developing limb bud, may drive chondrogenesis and could potentially be exploited for cartilage TE. However, as hypoxia also plays a role in both the formation and maintenance of cartilage, how hypoxia influences mechanoregulatory-mediated chondrogenesis is less well understood. This is particularly important as hypoxia and HIF have also been shown to directly influence the activity of both RhoA and ROCK [28], [29]. Moreover, how the presence of TGF-β_3_, which drives chondrogenic lineage specification, but may also have a mechanoregulatory role itself [27], [30], affects these processes is not well explored. We hypothesise that hypoxia affects the chondrogenic response of hMSC to soft substrates, and specifically, that a combination of soft culture substrates and hypoxia will promote upregulation of molecular markers of chondrogenesis and that these effects will be directed by actin-myosin tension.

To begin to address these questions, we cultured hMSC on fibronectin-coated PA substrates with Young’s moduli of either 0.167 (soft) or 49.6 kPa (stiff) in chondrogenic medium containing TGF-β_3_. We then compared cell morphology, phosphorylated myosin light chain 2 (pMLC2) staining, and gene expression in the presence and absence of hypoxia and in the presence and absence of pharmacological inhibition of cytoskeletal tension. Our findings show that on soft substrates, hypoxia prompts hMSC to adopt more spread morphologies, form mesenchymal condensation-like colonies, and upregulate *NCAM* expression, and that inhibition of cytoskeletal tension negates the hypoxia-mediated upregulation of chondrogenic gene expression. Indeed, our findings suggest that the effects of hypoxia may be, at least in part, dependent on mechanosensitive pathways.

## 2 Materials and methods

### 2.1 Human marrow stromal/mesenchymal stem cell (hMSC) isolation and culture

Human samples were obtained from the Imperial College Healthcare Tissue Bank (ICHTB, HTA license 12275). ICHTB are supported by the National Institute for Health Research Biomedical Research Centre at Imperial College Healthcare National Health Service Trust and Imperial College London. ICHTB is approved by the United Kingdom National Research Ethics Service to release human material for research (12/WA/0196). The samples for this project were issued from sub-collection R16052.

Bone marrow aspirates from healthy donors (collected with informed consent) were plated in CellSTACK® (Corning) culture chambers at a density of 10–25 × 10^6^/636 cm^2^. Cells were cultured in α-Minimal Essential Media (αMEM) supplemented with 5% human platelet lysate (Stemulate) under standard conditions (37 °C, 5% CO_2_/95% air). Upon confluence (10–14 days), cells were detached and frozen. Cells were then expanded in growth media (GM) consisting of αMEM with 10% (v/v) Foetal Bovine Serum (FBS; Life Technologies), which was replaced twice a week. Cells were passaged when they reached 90% confluency and used prior to passage 7. Cultures were immunophenotyped and found to express CD90, CD105, CD73 and not express hematopoietic markers CD34 and CD45 at passages 0, 4 and 7 (data not shown). Differentiation experiments were performed in chondrogenic differentiation media (CDM) consisted of High Glucose Dulbecco's Modified Eagle Medium (Sigma Aldrich) supplemented with 2 mM l-Glutamine (Thermo Fisher Scientific), 100 nM dexamethasome (Sigma Aldrich), 1% (v/v) Insulin, Transferrin, Selenium Solution (Thermo Fisher Scientific), 1% (v/v) antibiotic/antimycotic solution (Sigma Aldrich), 50 μg/ml ascorbic acid-2-phosphate (Sigma Aldrich), 40 μg/ml l-proline (Sigma Aldrich), and 10 ng/ml TGF-β_3_ (Peprotech).

### 2.2 Preparation and mechanical characterisation of polyacrylamide (PA) substrates

Polyacrylamide (PA) substrates were formed using a modified protocol described by Tse and Engler [31]. Briefly, 0.5 ml of 100 mM sodium hydroxide in deionised water (dH_2_O) was dispensed onto 25 mm glass coverslips and allowed to evaporate at 80 °C. 0.2 ml of (3-aminopropyl)triethoxysilane was then placed on coverslips and allowed to react for 5 min, washed thoroughly with deionised water (dH_2_O), and coverslips were placed in a 0.5% (v/v) glutaraldehyde solution in phosphate buffered saline (PBS) for 30 min before being allowed to air dry. 40% (w/v) acrylamide, 2% (w/v) *N*,*N*′-methylenebis(acrylamide), and PBS were mixed (Supplementary Table 1) and then degassed under vacuum for 15 min. Microscope slides were coated with 0.1 ml of dichlorodimethylsilane (DCDMS), allowed to react for 2 min and then washed with dH_2_O. Tetramethylethylenediamine (TEMED) and ammonium persulfate (APS) were added to gel precursors and vortexed for 30 s. The hydrogel solution was then dispensed onto DCDMS-coated microscope slides, coverslips placed onto the gel solution and allowed to cure for 30 min. Coverslips were then washed overnight in PBS with gentle agitation. Coverslips were washed 3 times PBS for 5 min each and a 0.5 mg/ml solution of sulfosuccinimidyl 6-(4′-azido-2′-nitrophenylamino)hexanoate in 50 mM HEPES (pH 8.5) was placed on coverslip surfaces and exposed to UV light for 20 min. Coverslips were then washed 3 times with HEPES and placed in a 0.015 mg/ml fibronectin solution in HEPES overnight at 4 °C. Fibronectin-coated substrates were washed 3 times in PBS and stored at 4 °C for up to 2 weeks.

The Young’s moduli (E) of PA gels on glass coverslips were characterised using atomic force microscopy (AFM) microindentation, using a modified protocol to that previously described [32]. Spherical glass beads (diameter 10 µm; Whitehouse Scientific) were mounted onto tipless triangular silicon nitride cantilevers (spring constant 0.12 N/m; Bruker AXS SAS) using UV cross-linked Loctite super glue. Cantilevers were then calibrated using thermal tuning to confirm the spring constant [33]. Force measurements were made on a Nanowizard 4 AFM with JPK SPM software 6.1 (JPK Instruments AG) in liquid in 10 areas on the surface of each gel (2 independent gels per formulation). Gels were indented 1–1.5 µm with a extend speed of 4 µm/s. E was then determined using JPK SPM software and fitted to the Oliver-Pharr model for a spherical tip [34], as previously described [35]. The Poisson’s ratio was assumed to be 0.5.

### 2.3 hMSC differentiation on PA substrates

PA hydrogels on coverslips were placed in 6-well plates prior to seeding. The appropriate number of hMSC were then resuspended in 200 μl of media and pipetted onto gels at a density of 3 × 10^4^ cells/cm^2^. Following an initial 4-hour attachment period, 2 ml of GM was added. After a further 24 h, GM was then replaced with CDM and maintained under either standard conditions or cultured an incubator set to provide 2% O_2_ (hypoxia) as indicated. CDM was supplemented with 10 μM Y-27632 (Merck Millipore) or 5 µM Blebbistatin (Sigma-Aldrich) as indicated.

### 2.4 Immunostaining and image quantification

Cultures were washed in PBS and fixed in 4% (w/v) paraformaldehyde for 15 min at room temperature (RT). Cultures were then placed in 5% (w/v) bovine serum albumin (BSA, Sigma Aldrich) in 0.1 M Triton X-100 in H_2_O (PBT) for 1 h at RT and then treated with primary antibodies (YAP, sc101199, Santa Cruz, 1:100; pMLC2, 3671S, Cell Signaling Technology 1:100; collagen type II, ab34712, Abcam 1:200) in 5% (w/v) BSA in PBT overnight at 4 °C. Cultures were washed in 3% (w/v) BSA in PBT with gentle agitation. Primary antibodies against pMLC2 and collagen type II were visualised after incubating with ab150077 (Abcam) for 1 h at RT (1:500 in 5% (w/v) BSA + PBT). Antibodies against YAP were detected using biotin conjugate secondary antibody (ab6788, Abcam) which were detected with a fluorescently labelled streptavidin biotin-binding protein (S11223, Thermo Fisher Scientific). The secondary antibody solution also included 0.1 μg/ml DAPI. Fluorescence signal was imaged on an Axiovert200M microscope (Zeiss). Following imaging, PA gels were re-stained with Alexa488 Phalloidin (Sigma Aldrich) at 1:200 in PBS for visualisation of the actin cytoskeleton.

### 2.5 Immunofluorescence quantification

Immunofluorescence images were captured on a Leica DM16000 confocal laser scanning microscope (Leica Microsystems) using identical gain, exposure and offset for all conditions in each experiment. These were determined with positive controls that expressed the antigen of interest, and negative controls in which the primary antibody was omitted (Supplementary Fig. 1). The same threshold fluorescence intensity for images of all conditions within an experiment was set and signal below this threshold was negated (ImageJ). Signal above the threshold was used to create binary representations of protein localisation and the percentage of immunofluorescence staining was then determined. Percentage was normalised to the number of DAPI-positive cells. To quantify colony area, phalloidin images were thresholded and made binary. Using the corresponding DAPI channel as a reference, colonies were counted based on classification of direct cell-cell contact (Supplementary Fig. 2). The area of single cells/colonies and single cell circularity were quantified using the ‘measure’ function in ImageJ. The number of cells per colony was calculated for each biological replicate by dividing the total number of cells within a colony by the total number of colonies for all images in each experimental condition. For quantification of YAP nuclear localisation, DAPI-stained nuclei were selected in each image and superimposed onto the corresponding Alexa488 channel. ImageJ’s ‘measure’ function was utilised to quantify the percentage of signal present within each DAPI-demarcated area in the Alexa488 channel.

### 2.6 Gene expression analyses

Samples were lysed in 1% (v/v) 2-mercaptoethanol in RLT buffer (Qiagen), stored at −80 °C and RNA was extracted using the RNeasy Mini Kit (Qiagen). RNA was eluted in RNase-free H_2_O and quantified on a NanoDrop spectrophotometer. 100 ng of RNA per sample was then reverse transcribed by incubating with Random Primers (Promega) at 70 °C for 5 min. cDNA was synthesised in 4% (v/v) Moloney Murine Leukemia Virus Reverse Transcriptase (MLV-RT; Promega) + 20% (v/v) MLV-RT buffer (Promega) + 5.4% (v/v) PCR Nucleotide Mix (Promega) all in molecular biology H_2_O (Sigma Aldrich) for 1 h at 42 °C. qPCR was carried out in a CFX384 (Biorad). qPCR reaction mixtures consisted of 4 ng cDNA + 50% (v/v) Brilliant III Ultra-Fast SYBR® Green QPCR Master Mix (Agilent) and primers (IDT Technologies) specific to genes of interest (Supplementary Table 2). Ct values were converted to transcript copy number by the relative standard curve method and expression levels normalised to transcript levels of *RPL13A*. Following normalisation to the housekeeping gene, expression levels were normalised to that of untreated controls to determine fold change. Each primer set produced a linear relationship between cDNA concentration and Ct value and reaction efficiencies were confirmed to be between 90 and 110%.

### 2.7 Statistical analyses

All statistical analyses were performed in Prism7 (GraphPad) with the Mann-Whitney test used to compare two conditions and Kruskal-Wallis with Dunn’s Correction for multiple condition comparisons, except for comparisons of single cells versus colonies, which were analysed using a Fisher’s exact test. Data comprise biological replicates (n stated in figure legends), where each replicate represents a single cell culture experiment.

## 3 Results

**Fig. 1:**
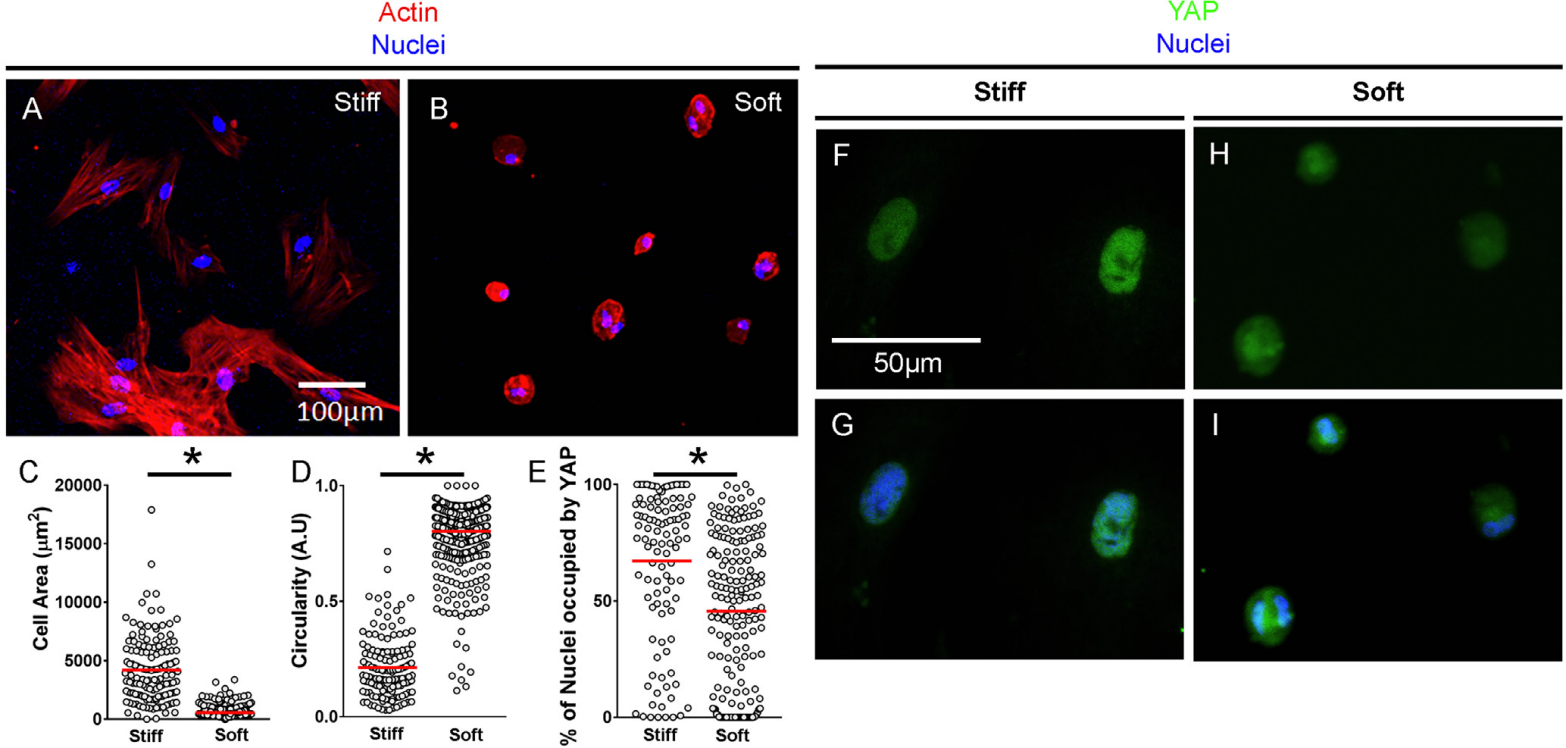
Substrate stiffness impacts cell morphology and YAP nuclear localisation in hMSC cultured in chondrogenic medium. (A + B) Actin immunodetection by phalloidin with DAPI counterstain after 24 h culture on stiff (A) and soft (B) substrates. Representative images of 4 independent repeats shown. (C + D) Quantification of cell area (C) and circularity (D) based on phalloidin staining. Values plotted represent the area/circularity of a single cell, with values from 4 independent repeats plotted and the mean values represented by the red line. A perfect circle has a circularity of 1. (E) Quantification of nuclear YAP on hydrogels of each stiffness. Each value plotted represents the percentage of a single DAPI-marked nucleus that is occupied by YAP. Values are from 3 independent repeats with the red horizontal lines representing the mean. (F–I) YAP immunodetection with DAPI counterstain after 24 h on stiff (F + G) and soft (H + I) substrates. Representative images of 3 independent repeats shown. Statistical analysis: ^*^p < 0.05. (For interpretation of the references to colour in this figure legend, the reader is referred to the web version of this article.)

hMSC are known to respond to culture on soft and stiff surfaces by altering their cytoskeletal arrangements [36], [37]. Here, we first aimed to confirm that under normoxic conditions in the presence of chondrogenic medium containing TGF-β_3_, substrate stiffness did indeed regulate hMSC spread area and circularity. To create culture substrates that cells would perceive as either akin to the soft matrix in the developing limb bud [38] or as significantly stiffer, we formed PA surfaces with varying concentrations of acrylamide and bis-acrylamide and measured their Young’s moduli using AFM-based microindentation (Supplementary Fig. 3). After coating with fibronectin, we then cultured hMSC for 24 h on the PA hydrogels and found that on stiff surfaces with a Young’s modulus of 49.6 kPa, they adopted spread morphologies and actin staining with fluorophore-conjugated phalloidin confirmed that they produced defined stress fibres (Fig. 1A). However, on soft surfaces with a Young’s modulus of 0.167 kPa, hMSC appeared round and stress fibres were not evident (Fig. 1B). Quantification showed that hMSC on stiff hydrogels had significantly larger cell areas (Fig. 1C) and that cells exhibited significantly lower levels of circularity compared to hMSC cultured on soft hydrogels (Fig. 1D).

Previous studies have shown that hMSC mechanosensing of substrate stiffness is mediated by the transcriptional co-activators of the hippo pathway YAP and TAZ [37]. Therefore, we stained hMSC for YAP to examine its intracellular localisation. On stiff surfaces, YAP was often confined to cell nuclei (Fig. 1F + G); however, on soft substrates it was more diffuse throughout the cytoplasm (Fig. 1H + I). Quantification of the percent of each DAPI-stained nucleus that was occupied by YAP, confirmed that on stiff surfaces, a greater percentage of nuclei were positive for the stain compared to on soft surfaces (Fig. 1E). These data are in line with previous observations [37] and confirm that hMSC cultured on stiff substrates in chondrogenic medium containing TGF-β3 adopt spread morphologies and YAP localises to cell nuclei, whilst on soft substrates, cells remain round and YAP is retained in the cytoplasm.

**Fig. 2:**
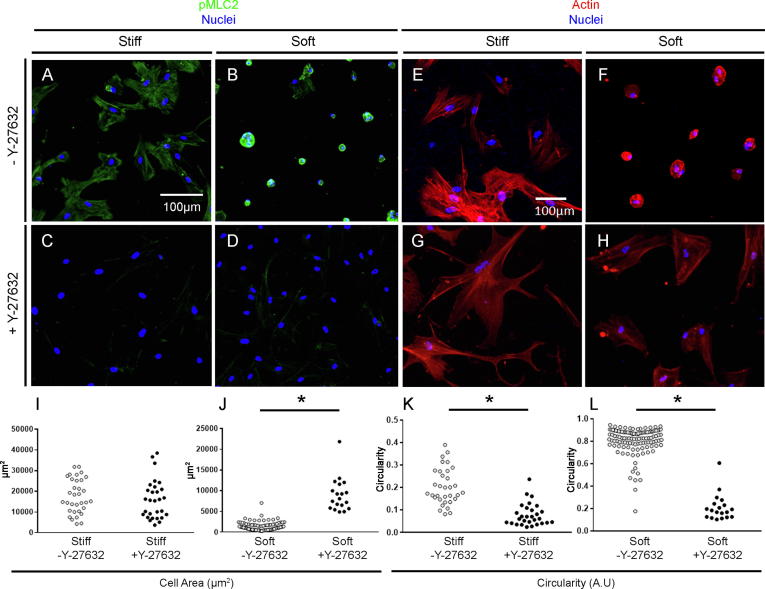
The ROCK inhibitor Y-27632 reduces myosin light chain 2 phosphorylation and induces differential effects on hMSC cytoskeletal arrangements on soft and stiff substrates. (A–D) pMLC2 immunodetection with DAPI counterstain after 24 h culture without Y-27632 on stiff (A) and soft (B) substrates and with Y-27632 (C + D). Representative images of 3 independent repeats shown. (E–H) Actin immunodetection by phalloidin with DAPI counterstain after 24 h of culture on stiff (E) and soft (F) substrates and with Y-27632 (G + H). (I + J) Quantification of single cell area based on phalloidin staining in E–H on stiff (I) and soft (J) with and without Y-27632. (K + L) Quantification of single cell circularity based on phalloidin staining in E–H on stiff (K) and soft (L) with and without Y-27632. A perfect circle has a circularity of 1. For direct comparisons of cell area and circularity on soft and stiff substrates, see Supplementary Fig. 4A–D. Statistical analysis: ^*^p < 0.05.

The RhoA/ROCK pathway is known to be a key mediator of cytoskeletal organisation and the cellular response to substrate stiffness [39]. To understand the role of ROCK activity in regulating hMSC morphology when cultured on soft and stiff hydrogels in chondrogenic medium, we next treated cells for 24 h with Y-27632, which binds the ATP-binding pocket of the catalytic site of RhoA targets ROCK1 and ROCK2. This inhibits the ability of ROCK1/ROCK2 to phosphorylate MLC2, thereby inhibiting actin-myosin tension [40]. hMSC on both soft and stiff hydrogels showed positive staining for pMLC2 (Fig. 2 A + B), which was abrogated by treatment with Y-27632 (Fig. 2C + D). Actin staining of hMSC on both soft and stiff surfaces similarly revealed changes in cell morphology in response to Y-27632. On stiff hydrogels, Y-27632 diminished pronounced stress fibre formation (Fig. 2E + G), as reported previously [41]. However, on soft hydrogels, Y-27632 prompted hMSC to adopt more spread morphologies (Fig. 2F + H). This was confirmed by quantitative analyses, as hMSC on soft surfaces treated with Y-27632 had both significantly larger spread areas and displayed lower levels of circularity compared to control conditions. This was in contrast with cell behaviour on stiff surfaces where spread area was not significantly affected by treatment with Y-27632, although hMSC did have significantly higher levels of circularity (Fig. 2I–L and Supplementary Fig. 4). Taken together, these data are in keeping with previous observations that Y-27632 inhibits ROCK activity, and thereby MLC2 phosphorylation, and prompts cells to adopt spread morphologies on soft substrates [42]. They also confirm that TGF-β_3_ does not hinder the effect of Y-27632, and demonstrate that under chondrogenic conditions, hMSC morphology on stiff and soft substrates is governed, at least in part, by ROCK. Our findings also demonstrate that in our hands, hMSC undergoing chondrogenic differentiation respond as expected to substrate stiffness and pharmacological disruption of cytoskeletal tension, providing us with a model to study how cellular mechanotransduction is impacted by hypoxia during TGF-β_3_-driven chondrogenesis.

**Fig. 3:**
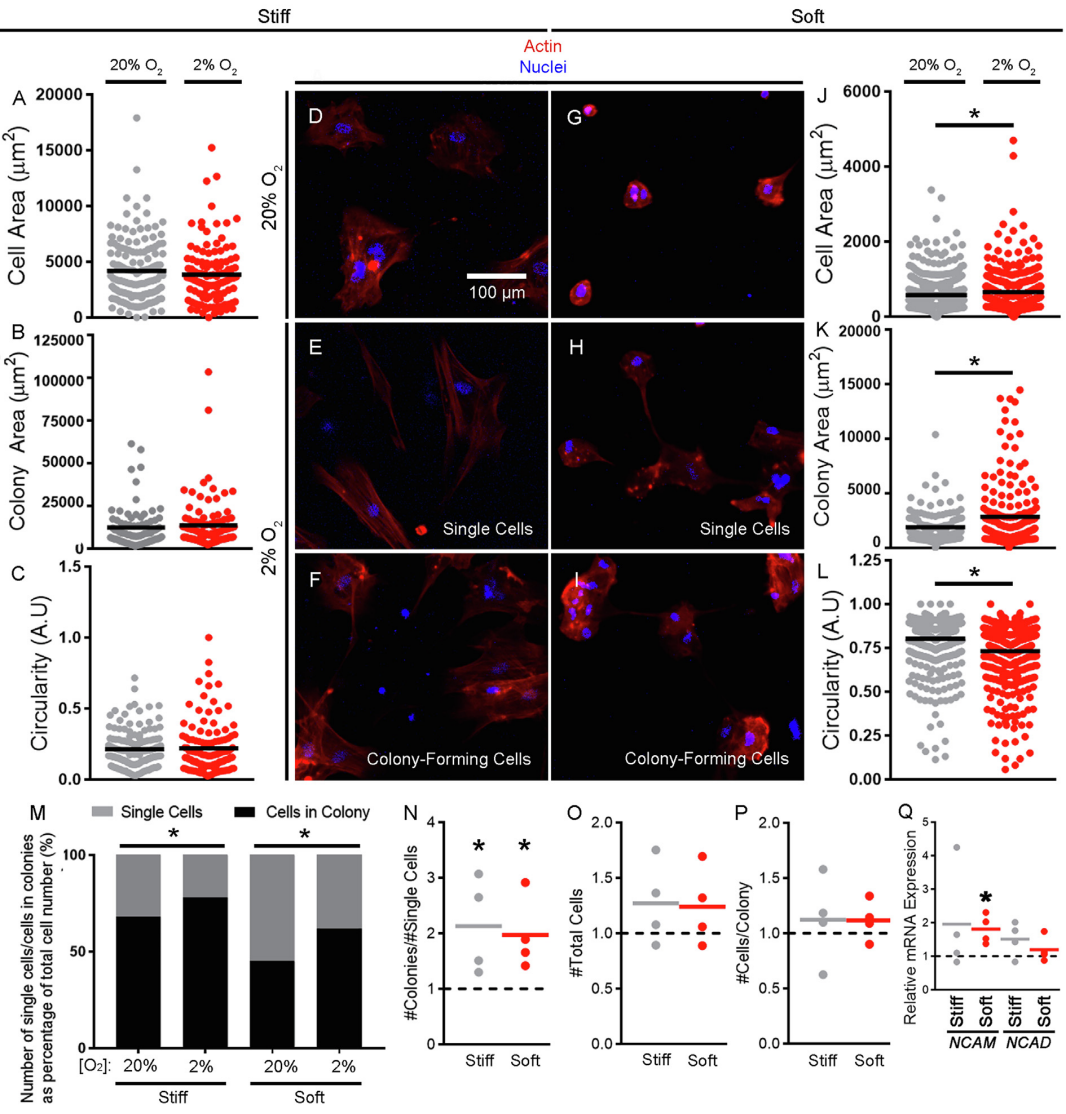
Hypoxia impacts hMSC morphology on soft substrates and increases colony formation. (A–C + J–L) Quantification of single cell area (A + J), colony area (B + K) and single cell circularity (C + L) based on phalloidin staining of hMSC on stiff and soft substrates under normoxic (20% O_2_) and hypoxic (2% O_2_) conditions. Values plotted represent the area/circularity of single cells/colonies from 4 independent repeats with the mean values represented by black lines. A perfect circle has a circularity of 1. (D–I) Actin immunodetection by phalloidin with DAPI counterstain after 24 h culture on stiff (D–F) and soft (G–I) substrates at 20% O_2_ (D + G) and 2% O_2_ (E, F, H, I). Representative images of 4 independent repeats shown. (M) Percentage of single or colony-forming cells on stiff and soft hydrogels at 20% and 2% O_2_ within each condition. Values plotted were calculated from the total number of observations (20% O_2_, soft, *n* = 844; 2% O_2_, soft, *n* = 1021; 20% O_2_, stiff, *n* = 408; 2% O_2_, stiff, *n* = 498) from 4 independent repeats. ^*^p < 0.001 when the number of colony-forming cells between normoxic and hypoxic conditions were compared. Quantification of colonies in response to 2% O_2_ normalised to single cell number (N), total cell number (O) and number of cells per colony (P) on stiff and soft. (Q) Expression of *NCAM* and *NCAD* in hMSC on soft and stiff in response to 2% O_2_. Values plotted are from 4 independent repeats and are fold change compared to that at 20% O_2_ on the respective substrate, represented by the horizontal dotted line, with grey/red lines representing the mean values. For direct comparisons of cell area and circularity on soft and stiff substrates, see Supplementary Fig. 4E–J. Statistical analysis: ^*^p < 0.05 (compared to the normoxic condition). (For interpretation of the references to colour in this figure legend, the reader is referred to the web version of this article.)

Hypoxia has been shown in both *in vitro* and *in vivo* models to regulate chondroprogenitor differentiation and cartilage formation [12], [43], and there is increasing evidence that it interacts with pathways governing cell response to mechanical cues such as substrate stiffness [28]. Therefore, we next examined how hMSC in chondrogenic medium responded to culture on soft and stiff hydrogels under hypoxic (2% O_2_) compared to normoxic (20% O_2_) conditions. We first observed that on stiff hydrogels, the single cell area as well as the circularity of hMSC stained with phalloidin did not change under hypoxic compared to normoxic conditions (Fig. 3A/C–E and Supplementary Fig. 4). This contrasted with culture on soft hydrogels where hypoxia prompted hMSC to adopt more spread morphologies with significantly larger cell areas and lower levels of circularity (Fig. 3G + H/J/L and Supplementary Fig. 4). In short, hypoxia impacted hMSC morphology in response to stiffness on soft, but not stiff substrates.

In addition to alterations in cell morphology on soft substrates, we also observed that on both soft and stiff substrates hMSC appeared to form colonies when cultured under hypoxic compared to normoxic conditions (Fig. 3F/I). Therefore, we first quantified colony area by identifying hMSC whose phalloidin positive area contacted at least 1 other cell, and found that on soft, but not stiff hydrogels, hypoxia did indeed prompt hMSC to form larger colonies (Fig. 3B/K). We then determined the percentage of cells in each condition that were either in colonies or as single cells (Fig. 3M). A larger percentage of hMSC on stiff substrates compared to soft substrates were in contact with at least one other cell (likely because of their larger cell areas) (Supplementary Fig. 5). However, on both soft and stiff surfaces, hypoxia significantly (*p* < 0.001) increased the percentage of cells in colonies (Fig. 3M) and the number of colonies normalised to the number of single cells (plotted relative to the normoxic condition (dotted line), Fig. 3N). These observations could not be explained by proliferation as neither hypoxia nor substrate stiffness significantly affected the number of hMSC on soft and stiff hydrogels (Fig. 3O). Instead our results suggest that not only did hypoxia result in hMSC on soft surfaces increasing their spread area, but it also prompted a greater fraction of hMSC to form colonies. However, although the number of cells per colony was no different on soft and stiff substrates (Fig. 3P), colony area was approximately 5 times lower on soft compared to stiff surfaces, yielding colonies on soft surfaces that were more compact.

NCAM and NCAD are known to play important roles in cell-cell adhesion and chondrogenesis during limb bud condensation [44], [45]. Therefore, we quantified mRNA encoding for both *NCAM* and *NCAD* in response to hypoxia on both soft and stiff substrates and observed that *NCAM* expression was significantly higher in hMSC cultured on soft hydrogels under hypoxic compared to normoxic conditions (Fig. 3Q). This contrasted with expression patterns on stiff hydrogels where hypoxia did not significantly affect *NCAM* transcript levels; and *NCAD* expression, which was not impacted by either substrate stiffness of hypoxia. These results indicate that not only does hypoxia influence cell spread area on soft surfaces, but it also promotes colony formation on both soft and stiff surfaces, which may be more conducive for chondrogenesis.

**Fig. 4:**
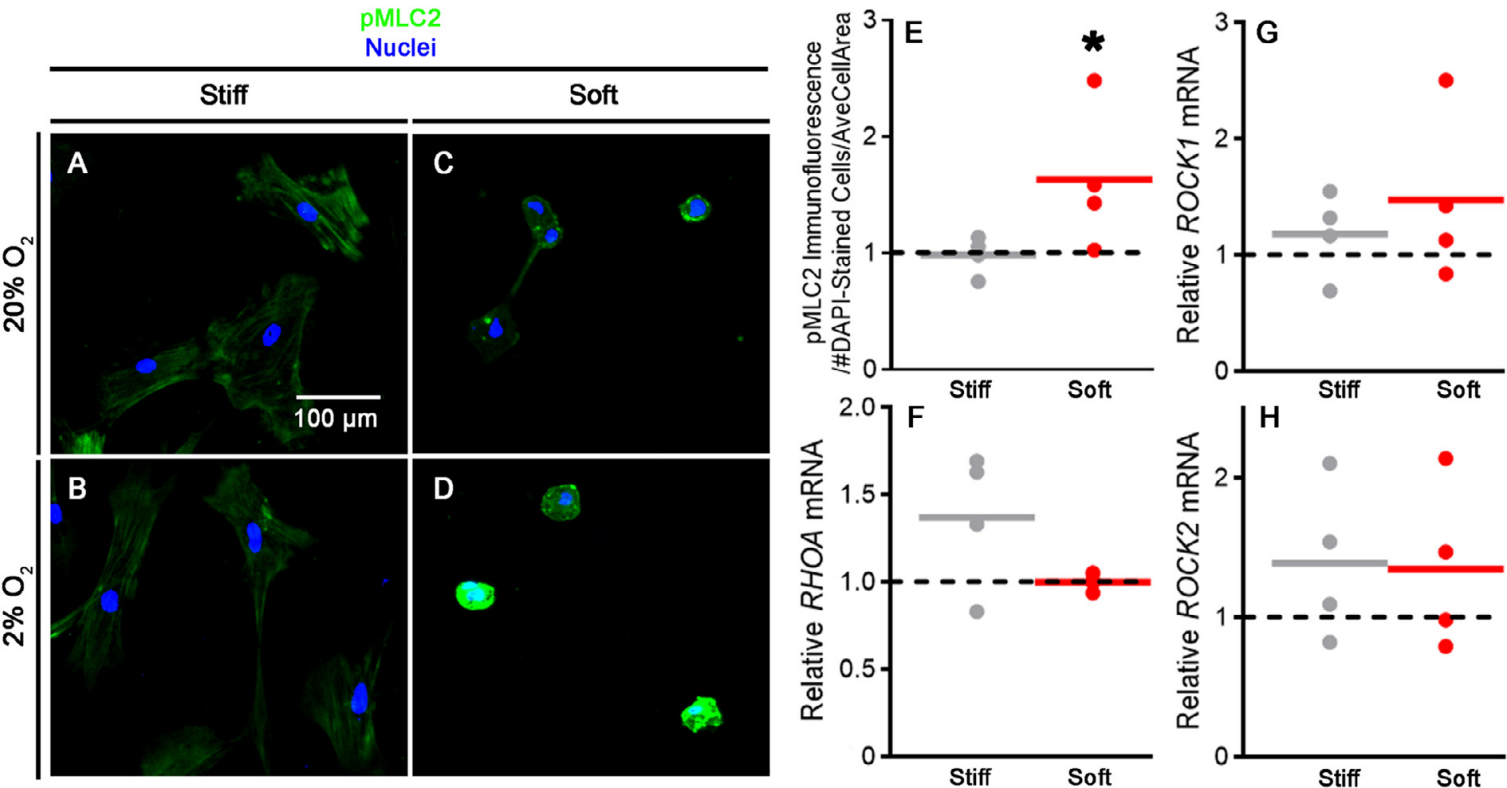
Hypoxia increases staining for phosphorylated myosin light chain 2 on soft substrates. (A–D) pMLC2 immunodetection with DAPI counterstain after 24 h at 20% O_2_ (A + C) and 2% O_2_ (B + D) on stiff (A + B) and soft (C + D) substrates. Representative images of 4 independent repeats shown. (E) Quantification of pMLC2 immunofluorescence in response to 2% O_2_ on stiff and soft substrates, normalised to DAPI-stained cell number and mean area of cells in each condition, calculated from Phalloidin images in Fig. 3D–I. (F–H) mRNA expression of *RHOA* (F), *ROCK1* (G) and *ROCK2* (H) following 24 h culture at 2% O_2_ on stiff and soft substrates. Values plotted in E–H are from 4 independent experiments and are fold change compared to pMLC2 immunofluorescence/mRNA expression at 20% O_2_ which is represented by the horizontal dotted line. Mean values are represented by grey/red lines. Statistical analysis: ^*^p < 0.05. (For interpretation of the references to colour in this figure legend, the reader is referred to the web version of this article.)

As hMSC cultured under chondrogenic conditions on soft hydrogels responded to hypoxia by increasing their cell area, and as hypoxia has been shown to influence both RhoA and ROCK activity [28], [29] by inducing cytoskeletal re-arrangement via actin-myosin-mediated contraction [17], [18], [27], [46], [47], [48], we next asked if hypoxia influenced pMLC-mediated cytoskeletal arrangements on soft and stiff substrates under hypoxic conditions, as phosphorylation of MLC2 is a molecular marker of ROCK activity. Oxygen concentration had no effect on staining for pMLC2 in hMSC cultured on stiff hydrogels (Fig. 4 A + B); however, on soft surfaces pMLC2 staining was enhanced under hypoxic conditions (Fig. 4C + D). These observations were confirmed by quantification of pMLC2 staining, which when normalised to cell number and average cell area, was significantly higher in response to hypoxia on soft but not stiff surfaces (Fig. 4E). Regulation of pMLC2 could not be attributed to increased transcriptional levels of either *RHOA*, *ROCK1* or *ROCK2*, as hypoxia did not significantly affect their expression (Fig. 4F–H), but rather suggests that increased pMLC2 staining may have been due to increased ROCK activity, perhaps via increased integrin expression [49]; however, further studies would be required to confirm this.

**Fig. 5:**
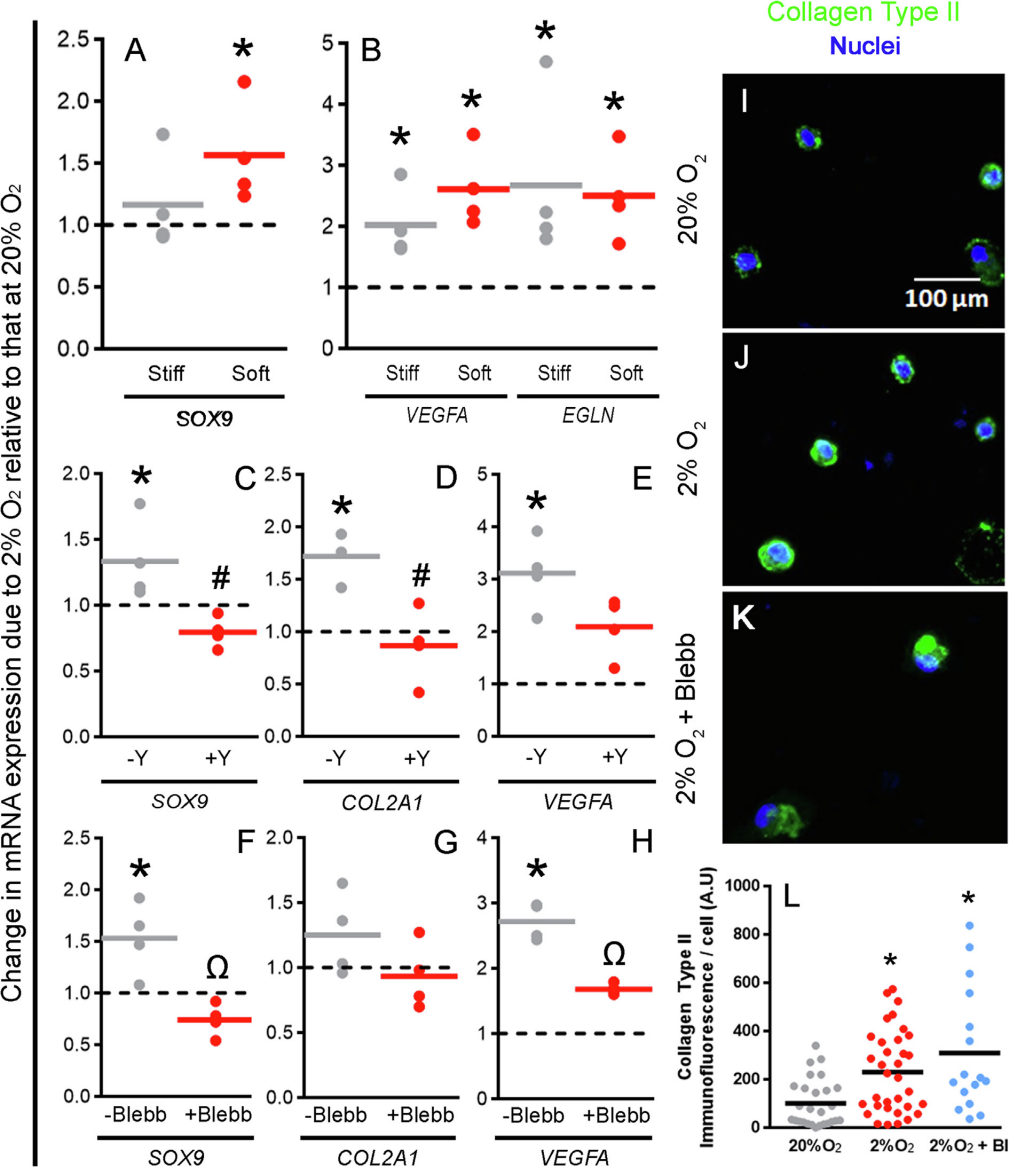
Hypoxia upregulates transcription of HIF target and early chondrogenic genes as well as immunodetection of collagen type II on soft substrates, which can be impacted by inhibitors of cytoskeletal tension. (A–B) Expression of mRNA encoding conserved HIF targets *VEGFA* and *EGLN* (A) and the chondrogenic transcription factor *SOX9* (B) following 24 h of culture at 2% O_2_ on soft and stiff substrates. (C–H) Expression of mRNA for *SOX9* (C), *COL2A1* (D) and *VEGFA* (E) following culture at 2% O_2_ on soft substrates without and with the ROCK inhibitor Y-27632 (−/+Y). (F–G) Expression of mRNA for *SOX9* (F), *COL2A1* (G) and *VEGFA* (H) following culture at 2% O_2_ on soft substrates without and with the inhibitor of MLC2 polymerisation Blebbistatin (−/+Blebb). Values plotted are from 4 independent experiments and are fold change compared to expression at 20% O_2_ represented by the horizontal dotted line. Mean values are represented by grey/red lines. (I–K) Collagen Type II immunofluorescence staining at day 14 of chondrogenesis of hMSC on soft substrates at 20% O_2_ (I), 2% O_2_ (J) and 2% O_2_ with Blebbistatin (K). Brightness and contrast were adjusted to an equal degree between all conditions. (L) Quantification of Collagen Type II immunofluorescence on a per cell basis (Bl = Blebbistatin). Black horizontal lines represent means for each condition. ^*^p < 0.05 compared to the 20% O_2_ condition. # represents a significant change (*p* < 0.05) between +/−Y conditions 2% O_2_ and Ω represents a significant change (*p* < 0.05) between +/−Blebb conditions at 2% O_2_. (For interpretation of the references to colour in this figure legend, the reader is referred to the web version of this article.)

As culturing hMSC on soft hydrogels under hypoxic conditions prompted changes in cell morphology and staining for pMLC2, we next asked if these conditions also affected molecular and protein markers of chondrogenesis and if this effect was facilitated by cytoskeletal tension. Hypoxia upregulated expression of *VEGFA* and *EGLN* (Fig. 5A), established targets of the HIF transcriptional complex. This observation confirmed that in our hands hMSC cultured on both soft and stiff surfaces in chondrogenic differentiation medium responded as expected to hypoxia. However, whilst on soft hydrogels, hypoxia significantly upregulated expression of HIF target gene, *SOX9* (Fig. 5B), on stiff surfaces, we could not detect a significant effect. The impact of hypoxia when hMSC were cultured on soft surfaces was also confirmed at the protein level. After 14 days in culture, hMSC cultured under hypoxic conditions showed a significant increase in immunostaining for collagen type II compared to cells cultured at normoxia (Fig. 5I–J, L).

We next asked if chondrogenic induction under hypoxic conditions on soft surfaces was mediated by ROCK signalling and found that treating cultures with Y-27632 abolished the hypoxia-mediated upregulation of *SOX9* (Fig. 5C), *COL2A1* (Fig. 5D) and *VEGFA* (Fig. 5E). This suggests that increased expression of HIF and chondrogenic markers on soft substrates in the presence of hypoxia is mediated, at least in part, by ROCK activity. In addition to ROCK activity, other mechanoregulatory pathways have also been shown to influence the cellular response to substrate stiffness. Therefore, we also treated hMSC with Blebbistatin, an inhibitor of MLC2, and observed that it also abolished the hypoxia-mediated upregulation of *SOX9* (Fig. 5F) and *VEGFA* (Fig. 5H) expression; however, we could not detect a significant effect on the expression of *COL2A1* (Fig. 5G) after 24 h or at the protein level after 14 days (Fig. 5K and L). Taken together, these data suggest that hypoxia plays in role in regulating hMSC mechanosensing during chondrogenic differentiation.

## 4 Discussion

Here, we confirmed that hMSC respond to culture on soft surfaces by adopting round morphologies and localising YAP diffusely in their cytoplasm, and that these effects are not grossly impacted in chondrogenic medium containing TGF-β_3_. We then showed that pharmacological inhibition of ROCK, which ablates positive staining for pMLC2, prompts hMSC on soft surfaces to adopt more spread morphologies. This is contrast to hMSC behaviour on stiff surfaces where treatment with Y-27632 only subtly affected cell morphology. These data suggest that on soft substrates, hMSC morphology is governed, at least in part, by ROCK, and confirm that chondrogenic induction with TGF-β_3_ does not impact hMSC’s expected behaviour under these conditions [42].

We then subjected hMSC to hypoxia and observed that on soft, but not stiff hydrogels, hypoxia impacts cytoskeletal arrangements and leads to increased staining for pMLC2. These observations suggest that hypoxic culture stimulates hMSC to increase their cytoskeletal tension and are in keeping with previous observations that inhibiting phosphatase activity increases myosin phosphorylation in a squamous cell carcinoma cell line [50]. The fact that hypoxia did not increase pMLC2 staining on stiff surfaces, however, should not be interpreted to mean that hypoxia does not impact pMLC2 more generally, as cytoskeletal tension on stiff substrates may have already been at maximum levels (hMSC are known to exert tension on stiff substrates [51]), and hMSC may not have been able to respond to additional stimuli. Moreover, we cannot rule out other effects associated with changes in cell area in response to either stiff surfaces or hypoxia. Indeed, Guo et al. have recently shown that cells reduce their volume in response to culture on stiff substrates, which may impact numerous cellular processes, including fate specification [52].

We found that hMSC responded to hypoxic conditions on soft hydrogels not only by changing their area, but also by aggregating within colonies. We could not explain our observations of colony formation by increased proliferation, suggesting that cells may have migrated towards one another to form condensations. Mesenchymal cells are known to be more migratory when cultured on soft compared to stiff surfaces [53]; however, our observation that this effect was enhanced under hypoxic conditions may suggest cross-talk between their respective mechanisms. Indeed, although we did not confirm this experimentally, one of the mechanisms by which hypoxia drives chondrogenesis on soft surfaces may be by enhancing cell-cell interactions. We observed that hMSC cultured on soft hydrogels under hypoxic conditions had increased expression levels of *NCAM*, but that *NCAD* expression was not affected. During *in vitro* chondrogenesis, NCAD is thought to initiate the formation of limb bud-like condensations, which NCAM then maintains [44], [45]. This suggests that in our model, hypoxia may not have initiated the condensations, but rather preserved cell-cell interactions. Moreover, as the total number of cells on both soft and stiff substrates was not affected by hypoxic culture, our observations of increased colony formation might suggest that cells rearranged themselves to increase cell-cell interactions. Taken together, these observations suggest that upregulation of *NCAM* may have played a role in regulating the increased expression of molecular markers for chondrogenesis we observed under these conditions, although further experiments would be necessary to confirm this role.

When cultured on soft surfaces, hypoxia upregulated hMSC chondrogenic gene/protein expression and this effect was, at least in part, mediated by ROCK activity. This was in agreement with others’ observations that hMSC express higher levels of *ACAN* and lower levels of hypertrophic markers *MMP13* and *COL10A1* when cultured within soft compared to stiff hyaluronic acid-based hydrogels [47]. They also support observations by Kim et al. who found that mouse chondrocytes cultured in soft collagen hydrogels upregulate expression of chondrogenic genes *COL2A1* and *ACAN* and downregulate expression of hypertrophic markers compared to that in chondrocytes cultured in stiff collagen hydrogels [20], and that this effect is mediated by myosin-mediated cytoskeletal tension, as treatment with Y-27632 or Blebbistatin could abolish it. Taken together, our data support previous reports that actin-myosin-mediated cytoskeletal tension is a driver of chondrogenesis, despite chondrocytes’ round morphologies, and further suggest that on soft surfaces, hypoxia can promote cytoskeletal tension, which further promotes chondrogenesis. They also raise the possibility that other cytoskeletal components that are known to be involved in chondrogenesis, including tubulin [54] and vimentin [55], may also play mechanosensory roles; however, additional experiments would be required to answer these questions. Moreover, our finding of enhanced hypoxia-mediated regulation of chondrogenic gene expression on soft surfaces is particularly interesting. Soft substrates and round hMSC morphologies seemed to promote hypoxia-mediated chondrogenic differentiation whereas on stiff substrates, hypoxia’s chondrogenic effect appeared to be moderated. This finding is in keeping with observations that round cell shapes in hypoxic areas of the developing limb bud correlate with chondrogenesis [15].

Despite our observations, however, colony formation under hypoxic conditions itself may also play an important role in stimulating chondrogenesis. Pellet culture has long been known to promote chondrogenesis [56]. However, whilst hypoxia stimulated colony formation on both soft and stiff hydrogels, colonies on soft hydrogels were far more compact that those on stiff (∼5 times smaller). These observations suggest that compact colony formation on soft substrates under hypoxic conditions could have contributed to the upregulation of chondrogenesis, as has been previously described [57]. These data suggest that the soft, low oxygen conditions reminiscent of the local milieu in which limb bud progenitors condense and differentiate into chondrocytes in the developing growth plate may also stimulate hMSC chondrogenesis *in vitro*.

Although we identified a role for mechanoregulation of hMSC morphology and chondrogenic differentiation under hypoxic conditions on soft hydrogels, other signalling pathways may also play a role. TGF-β1 is known to upregulate RhoA expression and prompt actin reorganisation via a SMAD2/3 dependent mechanism [30]. Similarly, Park et al. have shown that adding TGF-β1 to hMSC cultures on stiff surfaces enhances their contractile phenotype, but on soft surfaces, it does not [27]. Moreover, on soft surfaces, the addition of TGF-β1 increases expression of *COL2A1*. These observations confirm that increasing cytoskeletal tension on soft surfaces enhances the chondrocyte phenotype and are in keeping with our observations that increasing cytoskeletal tension via hypoxia promotes hMSC differentiation down the chondrogenic lineage.

Our observations may have important implications for cartilage TE strategies. We found that hypoxia upregulates chondrogenesis to a greater extent when cells are cultured on surfaces with a Young’s modulus of 0.167 compared to 49.6 kPa. We were unable to untangle whether this was mediated via a pure mechanoregulation-based mechanism or more indirectly through the formation of compact condensation-like colonies; nevertheless, our findings suggest that soft TE scaffolds may better enhance chondrogenesis under hypoxic conditions than that which is possible on stiffer scaffolds. Studies have suggested that for hMSC to appropriately differentiate along a particular lineage, they should be cultured on surfaces that match the mechanical stiffness of the native tissue [36]. Here, the Young’s modulus of the soft surfaces we cultured hMSC on were far softer than native cartilage, but were closer to that postulated for the developing limb bud [38]. This suggests that for cartilage TE, scaffolds may function best if they mimic not the stiffness of the native tissue, but rather that of the developing tissue. As cartilage is, for the most part, only formed during development, it may be that its transitory mechanical properties are those which TE strategies should aim to mimic.

## 5 Conclusions

Our findings show that on soft substrates hypoxia prompts cells to adopt more spread morphologies, assemble in compact mesenchymal condensation-like colonies, and upregulate *NCAM* expression and that these processes are, at least in part, dependent on cytoskeletal tension. Understanding the factors that regulate chondrogenic differentiation of hMSC may inform on strategies to repair acute cartilage defects using TE approaches. Indeed, our findings suggest that soft TE scaffolds that mimic the soft conditions that progenitor cells experience during native tissue formation may be more conducive for driving hypoxia-mediated chondrogenesis than stiffer scaffolds.
