# Supplementary material for: Hypoxia impacts human MSC response to substrate stiffness during chondrogenic differentiation
Source: Acta Biomater. 2019 Apr 15;89:73–83. doi: 10.1016/j.actbio.2019.03.002 (PMC6481516; doi:10.1016/j.actbio.2019.03.002)
Supplement: Supplementary data 1 [file mmc1.docx]

**Supplementary Table 1**: Components combined to form soft and stiff polyacrylamide substrates and measured Young’s modulus (E) in kPa.

|  | **Acrylamide (μl)** | **Bis-acrylamide (μl)** | **PBS**  **(μl)** | **APS (μl)** | **TEMED (μl)** | **E**  **(kPa)** |
| --- | --- | --- | --- | --- | --- | --- |
| **SOFT** | 75 | 30 | 895 | 10 | 1 | 0.167 |
| **STIFF** | 200 | 240 | 560 | 10 | 1 | 49.6 |

**Supplementary Table 2**: Forward and reverse primer sequences and primer concentration used for qPCR analyses.

| **Gene of Interest** | **Forward Primer Sequence** | **Reverse Primer Sequence** | **Concentration (nM)** |
| --- | --- | --- | --- |
| *VEGFA* | AGGGCAGAATCATCACGAAGT | AGGGTCTCGATTGGATGGCA | 250 |
| *RHOA* | AGCCTGTGGAAAGACATGCTT | TCAAACACTGTGGGCACATAC | 500 |
| *ROCK1* | AACATGCTGCTGGATAAATCTGG | TGTATCACATCGTACCATGCCT | 250 |
| *ROCK2* | TCAGAGGTCTACAGATGAAGGC | CCAGGGGCTATTGGCAAAGG | 500 |
| *NCAM* | GGCATTTACAAGTGTGTGGTTAC | TTGGCGCATTCTTGAACATGA | 500 |
| *NCAD* | TCAGGCGTCTGTAGAGGCTT | ATGCACATCCTTCGATAAGACTG | 250 |
| *RPL13A* | GCCATCGTGGCTAAACAGGTA | GTTGGTGTTCATCCGCTTGC | 250 |
| *EGLN* | AGGCGATAAGATCACCTGGAT | TTCGTCCGGCCATTGATTTTG | 250 |
| *SOX9* | AGCGAACGCACATCAAGAC | CTGTAGGCGATCTGTTGGGG | 250 |
| *COL2A1* | CCAGATGACCTTCCTACGCC | TTCAGGGCAGTGTACGTGAAC | 500 |


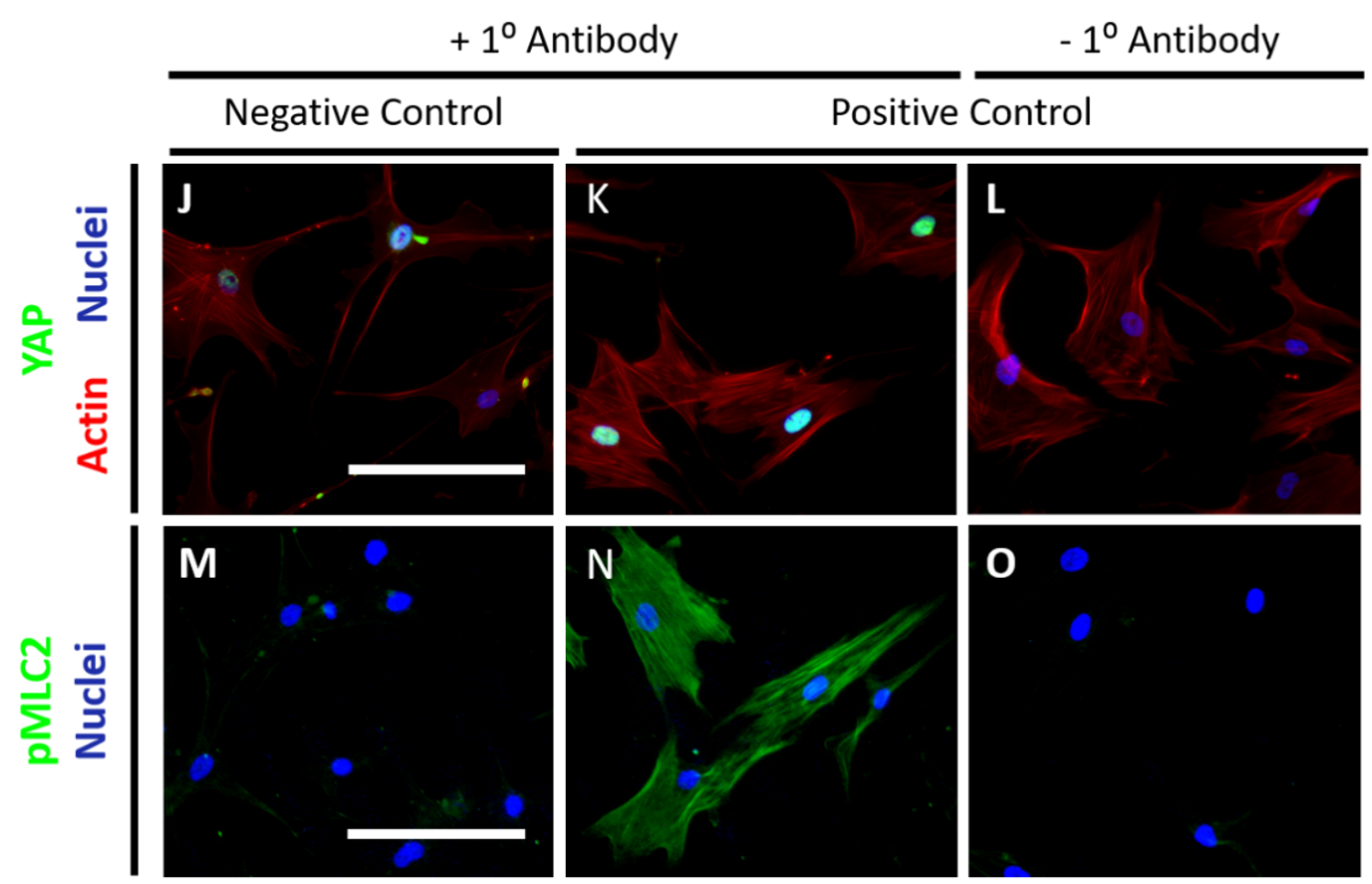


**Supplementary Figure 1**: Confirmation of YAP and pMLC2 antibody specificity. Scale bar = 200 µm.


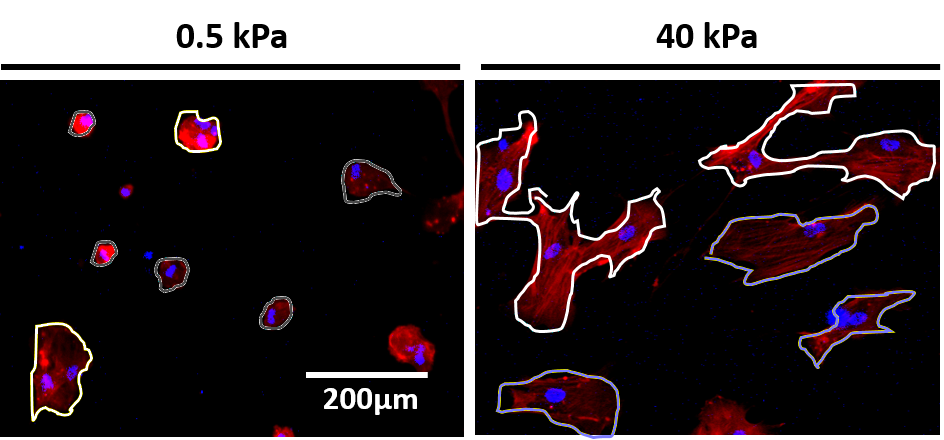


**Supplementary Figure 2**: Example classification of colonies and single cells on PA hydrogels. Single cells are outlined in grey and colonies are outlined in white.

**

**

**Supplementary Figure 3:** Young’s modulus (E, kPa) of soft, 75/30 and stiff, 200/24 (acrylamide/bis-acrylamide) hydrogels as determined by atomic force microscopy (AFM) microindentation. Soft hydrogels had a median E of 167 Pa and stiff hydrogels produced a median E of 49.6 kPa. Data comprise 1151 measurements on 2 independent soft gels and 1169 measurements on 2 independent stiff gels.


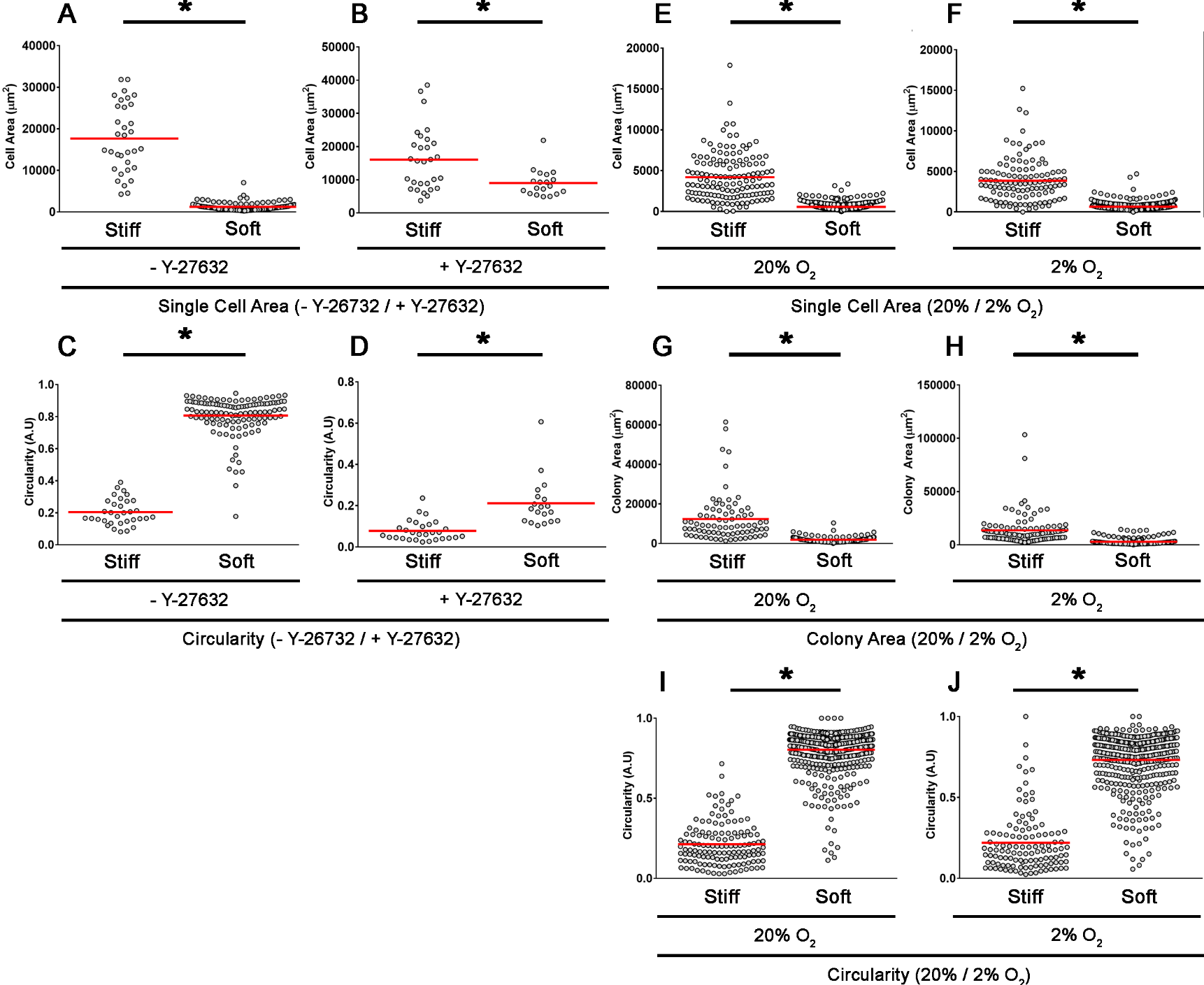


**Supplementary Figure 4**: Quantitative analyses of the effect of substrate stiffness on hMSC cell area, circularity and colony area after 24 h culture in chondrogenic medium under normoxic and hypoxic conditions and in the presence and absence of the ROCK inhibitor, Y-27632. Quantification of phalloidin-stained actin to determine single cell area (A+B, E+F), colony area (G+H) and circularity (C+D, I+J) under normoxic (E, G, I) and hypoxic (F, H, J) conditions. Panels A+C show cells response without, and B+D with the addition of Y-27632. Values plotted are from 4 independent repeats. Mean values are represented by the red line. Representative images for panels A-D are shown in Figure 2, and representative images for panels E-J are shown in Figure 3. **p* < 0.05.


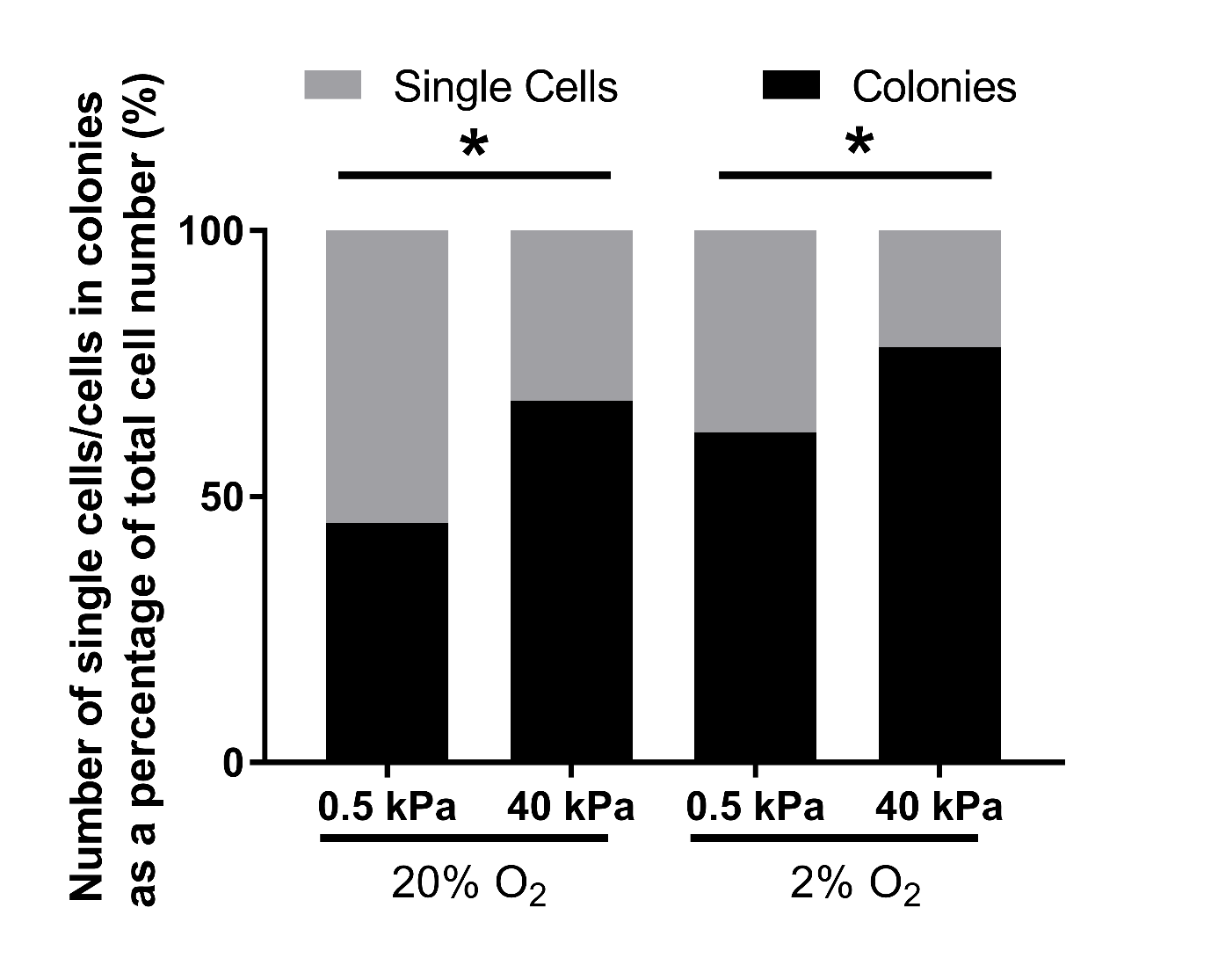


**Supplementary Figure 5**: Percentage of colony-forming cells on stiff and soft hydrogels at 20% and 2% O_2_. Values plotted were calculated from the total number of observations (20% O_2_, soft, n = 844; 2% O_2_, soft, n = 1021; 20% O_2_, stiff, n = 408; 2% O_2_, stiff, n = 498) from 4 independent repeats. **p* < 0.05 when the number of colony-forming cells between stiff and soft conditions were compared for both 20% and 2% O_2_ conditions.
